# mRNAs encoding neurodevelopmental regulators have equal N6-methyladenosine stoichiometry in Drosophila neuroblasts and neurons

**DOI:** 10.1186/s13064-022-00166-4

**Published:** 2022-10-15

**Authors:** Josephine D. Sami, Robert C. Spitale, Michael D. Cleary

**Affiliations:** 1 Department of Molecular and Cell Biology, Quantitative and Systems Biology Graduate Program, University of California, Merced, CA USA; 2 Department of Pharmaceutical Sciences and Department of Chemistry, University of California, Irvine, CA USA

## Abstract

N^6^-methyladenosine (m6A) is the most prevalent internal mRNA modification in metazoans and is particularly abundant in the central nervous system. The extent to which m6A is dynamically regulated and whether m6A contributes to cell type-specific mRNA metabolism in the nervous system, however, is largely unknown. To address these knowledge gaps, we mapped m6A and measured mRNA decay in neural progenitors (neuroblasts) and neurons of the *Drosophila melanogaster* larval brain. We identified 867 m6A targets; 233 of these are novel and preferentially encode regulators of neuroblast proliferation, cell fate-specification and synaptogenesis. Comparison of the neuroblast and neuron m6A transcriptomes revealed that m6A stoichiometry is largely uniform; we did not find evidence of neuroblast-specific or neuron-specific m6A modification. While m6A stoichiometry is constant, m6A targets are significantly less stable in neuroblasts than in neurons, potentially due to m6A-independent stabilization in neurons. We used in vivo quantitative imaging of m6A target proteins in *Mettl3* methyltransferase null brains and *Ythdf* m6A reader overexpressing brains to assay metabolic effects of m6A. Target protein levels decreased in *Mettl3* null brains and increased in *Ythdf* overexpressing brains, supporting a previously proposed model in which m6A enhances translation of target mRNAs. We conclude that m6A does not directly regulate mRNA stability during *Drosophila* neurogenesis but is rather deposited on neurodevelopmental transcripts that have intrinsic low stability in order to augment protein output.

**Supplementary Information:**

The online version contains supplementary material available at 10.1186/s13064-022-00166-4.

## Background

N^6^-methyladenosine or “m6A” is the most common nucleotide modification within eukaryotic mRNAs. This epitranscriptome mark is recognized by reader proteins that affect multiple mRNA metabolic processes, including splicing, decay and translation [1]. m6A is highly enriched in the nervous system of multiple organisms, including mammals, and has been implicated in events ranging from neural stem cell differentiation [2] to synaptic plasticity [3]. While multiple lines of evidence support the importance of m6A in neural development, a comprehensive understanding of neurodevelopmental processes affected by m6A is still lacking. In particular, whether or not m6A targets and the metabolic effects of m6A vary by neural cell type or neurodevelopmental stage is largely unknown. This information is important for determining the degree to which m6A influences the diversity of cellular fates and functions in the nervous system.

In mammals, cytoplasmic m6A is primarily found in the 3’UTR or at stop codons and is recognized by three readers: YTHDF1, YTHDF2, and YTHDF3. Early work assigned distinct roles to each reader (YTHDF1 and 3 promote translation, YTHDF2 promotes mRNA degradation) and suggested that each “DF” protein bound distinct mRNAs [4]. However, recent studies strongly suggest that all DF proteins target the same set of mRNAs and act redundantly via a single mechanism to induce mRNA decay [5]. There may be exceptions to this rule; for example, rare 5’UTR m6A promotes translation by directly recruiting the initiation factor eIF3 [6]. Dynamic regulation of m6A target metabolism could conceivably occur via variation in m6A stoichiometry (the fraction of transcripts that contain the modification), but quantitative analyses of m6A across cell types supports a model in which m6A targeting and frequency is uniform regardless of cell type or physiology [4].

Here we investigate m6A dynamics within the developing central nervous system of *Drosophila melanogaster*. *Drosophila* provides multiple advantages for m6A research: m6A is present at high levels in the embryonic, larval and adult nervous system; deletion of the *Mettl3* methyltransferase gene is not lethal, thus allowing molecular and phenotypic analyses in m6A-null animals; and the *Drosophila* genome encodes a single cytoplasmic reader, Ythdf, simplifying experiments aimed at manipulating the m6A system. The m6A methyltranscriptome has previously been mapped in *Drosophila* cell lines [7], embryos [8], and adult heads [9]. Multiple genetic approaches have demonstrated that m6A is involved in *Drosophila* sex determination [10], locomotion [11], learning and memory [9], and axon growth [7]. As in mammals, several molecular mechanisms have been assigned to m6A in *Drosophila*. In the nucleus, m6A regulates splicing [10] and m6A at the 5’ end of nascent transcripts relieves RNA polymerase II pausing to promote transcription [12]. In mature cytoplasmic transcripts, m6A is almost exclusively found in the 5’ UTR (in contrast to the 3’ UTR and stop codon localization found in mammals). *Drosophila* 5’ UTR m6A is thought to affect translation in one of two ways. First, m6A *decreases* translation of a subset of targets that are bound, in a Ythdf-dependent manner, by the translation repressor Fmr1 [7]. Second, 5’ UTR m6A has been shown to *increase* translation based on reporter assays and the observation that *Mettl3* loss-of-function causes a widespread decrease in nascent protein production [9]. 5’ UTR m6A is enriched among transcripts with low translation efficiency and Kan et al. proposed a model in which an m6A-dependent mechanism counteracts inefficient translation to augment target protein production [9].

While previous work in *Drosophila* identified m6A targets and molecular mechanisms, several knowledge gaps remain, especially with respect to neural development. First, it is unclear to what degree prior m6A mapping efforts identified targets relevant to neural progenitors; previous mapping in embryos included all cell types (of which neural progenitors are a tiny fraction) and adult heads lack neural progenitors. Second, while prior work ruled out a correlation between m6A and mRNA decay [9], this was based on aligning adult head m6A targets with embryonic central nervous system half-life data; m6A targets and mRNA half-lives were not compared in equivalent neural cell populations. Finally, experiments aimed at measuring the effects of m6A on target protein output in the nervous system, in vivo, are lacking and could help identify mechanisms relevant to specific neural cell types.

This work addresses the above knowledge gaps by obtaining methyltranscriptome maps that are representative of the neural progenitor and neuron populations in the *Drosophila* larval brain. The larval brain contains a well-defined population of neural stem cells, called neuroblasts, that undergo multiple rounds of asymmetric self-renewing divisions to ultimately produce neurons and glia. Using genetic manipulation and RNA profiling techniques, we obtained neurodevelopmental m6A maps that allowed comparisons of m6A stoichiometry between neuroblasts and neurons as well as investigation of how m6A influences mRNA stability in neuroblasts and neurons. We found extensive m6A targeting of neurodevelopmental regulators, including m6A modification of progenitor-specific transcripts. However, among transcripts expressed in both neuroblasts and neurons, we did not find any evidence of differential m6A stoichiometry. We confirmed the previously described correlation between m6A and translation efficiency and found a neuroblast-specific correlation between m6A and mRNA decay. Finally, we used in vivo imaging to demonstrate that m6A enhances target protein output in neuroblasts and neurons. Our findings support a model in which m6A is uniformly deposited on neurodevelopmental transcripts with intrinsic low stability and low translation efficiency and serves to augment protein production from those target mRNAs.

## Methods

### Drosophila genetics

The following lines were obtained from the Bloomington *Drosophila* Stock Center: Oregon-R-P2 (wildtype) (stock # 2376), *insc-Gal4* (stock # 8751), *nSyb-Gal4* (stock #51635), and *UAS-CD:UPRT* (stock # 77120). *UAS-aPKC*^*CAAX*^ was a gift from C.Y. Lee. *UAS-Ythdf* and *Mettl3* null flies were gifts from E. Lai.

### meRIP

m6A-RNA immunoprecipitation was performed as previously described [13]. Biological replicate experiments were performed for all three genotypes (wildtype, *insc* > *aPKC*^*CAAX*^*,* and *Mettl3* null)*.* Purified m6A-RNA was used to make sequencing libraries using the NuGen Ovation Universal RNA-Seq protocol, including adapter ligation and ribosomal RNA depletion using a *Drosophila*-specific AnyDeplete rRNA primer mixture. Libraries were amplified and purified according to the NuGen protocol and quality was assessed using an Agilent Bioanalyzer DNA high-sensitivity chip.

### EC-tagging pulse-chase

5-ethynylcytosine was synthesized as previously described [14]. Biological triplicate samples were prepared by carrying out 5EC feeding and RNA processing independently. Larvae were reared at 25 °C and fed 1 mM 5EC from 72 – 84 h after hatching prior to RNA extraction (pulse samples) or transferred to media with 10 mM uridine for 3, 6, or 12 h prior to RNA extraction (chase samples). Crudely dissected central nervous system RNA was extracted using Trizol. For each genotype and timepoint, duplicate 20 mg RNA samples were biotinylated using Click-iT Nascent RNA Capture reagents (ThermoFisher), purified on Dynabeads MyOne Streptavidin T1 magnetic beads (ThermoFisher) and used for “on bead” RNA-seq library synthesis, as previously described [15].

### RNA-sequencing and bioinformatics

Sequencing was performed on a HiSeq 2500. Sequence data were pre-processed with *FastQC*. Reads were trimmed using Trimmomatic to discard any reads with adaptor contamination and low-quality bases. We used *STAR* to map reads to the Ensembl gene annotation for *Drosophila melanogaster* (BDGP6). Peaks were identified by running MACS2 [16] with default parameters. For input RNA-seq and pulse-chase RNA-seq, reads were mapped using *kallisto* [17]. meRIP-seq data were quantified and mapped using *featureCounts* and those data were used in differential expression analysis with *limma-voom* [18]. *Limma-voom* was used to identify genes with significantly higher meRIP-seq counts in wildtype brains compared to *Mettl3* null brains. All candidates that lacked significant counts above *Mettl3* null were visually inspected in IGV to determine if the gene should be considered a m6A target. *PeakAnnotator* was used to annotate m6A position, as previously described [19]. Gene ontology analysis was performed using GO TermFinder [20] with the full *Drosophila melanogaster* gene set as background and default settings.

### RT-qPCR

First strand cDNA was made using the SuperScript VILO cDNA Synthesis Kit (Invitrogen). Real-time PCR quantitation was performed on a Rotor-Gene Q (Qiagen) in 20 mL reactions using SYBR green detection. Custom PCR oligonucleotides (Integrated DNA Technologies) were used for all targets: *run* forward (TAGGACAAAGGACCCCAATC), *run* reverse (TCGTCGCACGATTTTATGAG), *Sp1* forward (TTGAAGCTATCTTGCGGTTG), *Sp1* reverse (ATAGAGCGGGCGTTTCTTTC), *5S rRNA* forward (GCCAACGACCATACCACGCT), *5S rRNA* reverse (AGGCCAACAACACGCGGTATTCCCA)*.* Triplicate RT-qPCR experiments (starting at the m6A immunoprecipitation step) were performed for all target transcripts.

### Imaging and quantification of target proteins

The following antibodies were used: guinea pig anti-Runt (gift of C. Desplan) at 1:400, rabbit anti-CycD (Santa Cruz Biotech, sc-25765) at 1:250, and rabbit anti-Ase (gift of Y.N. Jan) at 1:1,000. Alexa-fluor conjugated secondary antibodies (ThermoFisher) were used. Brain imaging was performed using a Zeiss LSM 880 confocal microscope. Immunostaining was performed in parallel for all targets and genotypes with confocal settings kept constant. Pixel intensity measurements were made using ImageJ and the ‘‘measure’’ tool applied to an identical size area of interphase nuclei of neuroblasts, individual neurons, and multiple brain regions lacking expression of the protein of interest to calculate background signal.

## Results

### Transcripts encoding neurodevelopmental regulators are m6A modified in neuroblasts and neurons

**Fig. 1:**
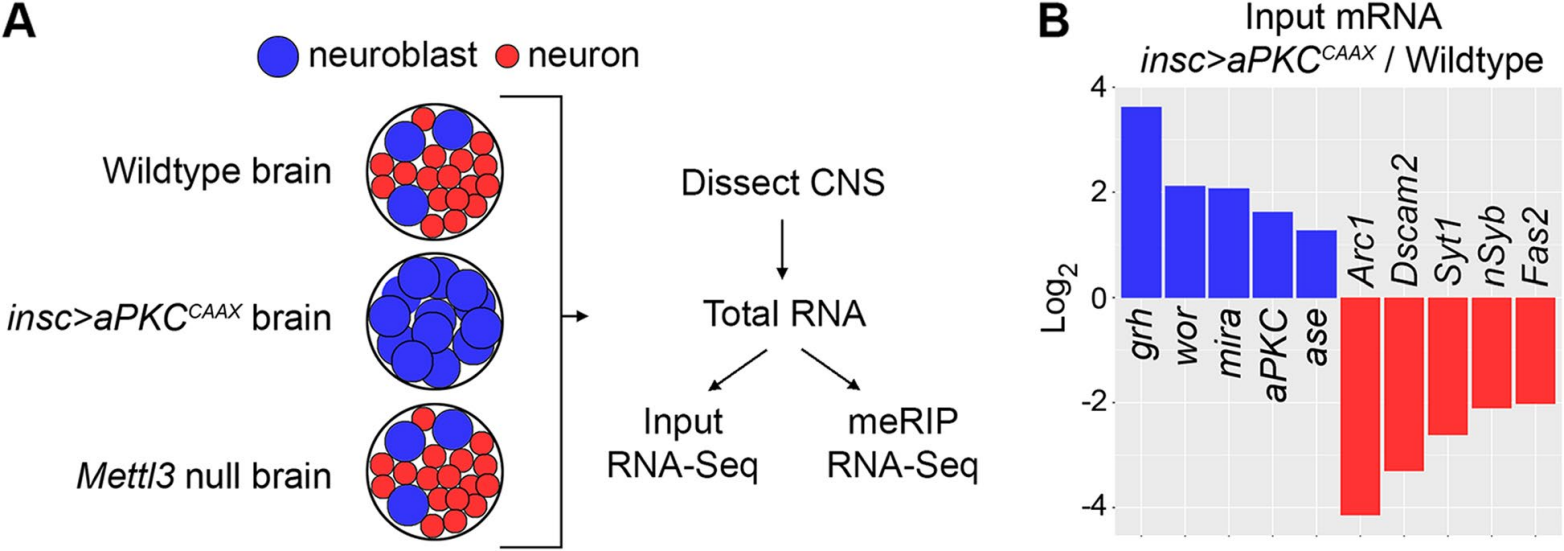
Confirmation of neuroblast-biased and neuron-biased transcriptomes. **A** Summary of experimental design. **B** Relative abundance of known neuroblast-specific mRNAs (blue) and known neuron-specific mRNAs (red) in insc > aPKCCAAX vs. wildtype brains. Average fold-change is shown

Near the end of *Drosophila* larval neurogenesis, the combined brain lobes contain approximately 10,000 neurons, roughly 500 glia, and only 200 neuroblasts [21, 22]. To increase representation of the neuroblast methyltranscriptome, we used a genetic modification that causes neuroblasts to undergo symmetric self-renewing divisions, thus generating larval brains with abundant ectopic neuroblasts and relatively few neurons [21]. In these experiments, we used *insc-Gal4* to drive expression of *UAS-aPKC*^*CAAX*^ in neuroblasts and harvested larval brains at 96—102 h after larval hatching (ALH) as a source of “neuroblast-biased RNA”. In contrast, we used wildtype larval brains at 96—102 h ALH as a source of “neuron-biased RNA” since neurons are vastly more abundant than any other cell type at this stage. In addition to collecting RNA samples that cover the neuron and neuroblast methyltranscriptomes, we collected RNA from stage-matched brains of *Mettl3* null larvae to obtain negative control “m6A null RNA”. Brain RNA from each genotype was split in two; half was used for quantification of total mRNA abundance (input RNA-seq) and half was used for methyltranscriptome purification using anti-m6A immunoprecipitation (meRIP-seq) [13]. This experimental design is summarized in Fig. 1A. As a first step, we used input RNA to test for differential abundance of known neuroblast or neuron-specific mRNAs in the neuron-biased and neuroblast-biased samples. We confirmed that *insc-Gal4* > *UAS-aPKC*^*CAAX*^ samples are enriched in neuroblast-specific transcripts and depleted of neuron-specific transcripts (Fig. 1B).

**Fig. 2:**
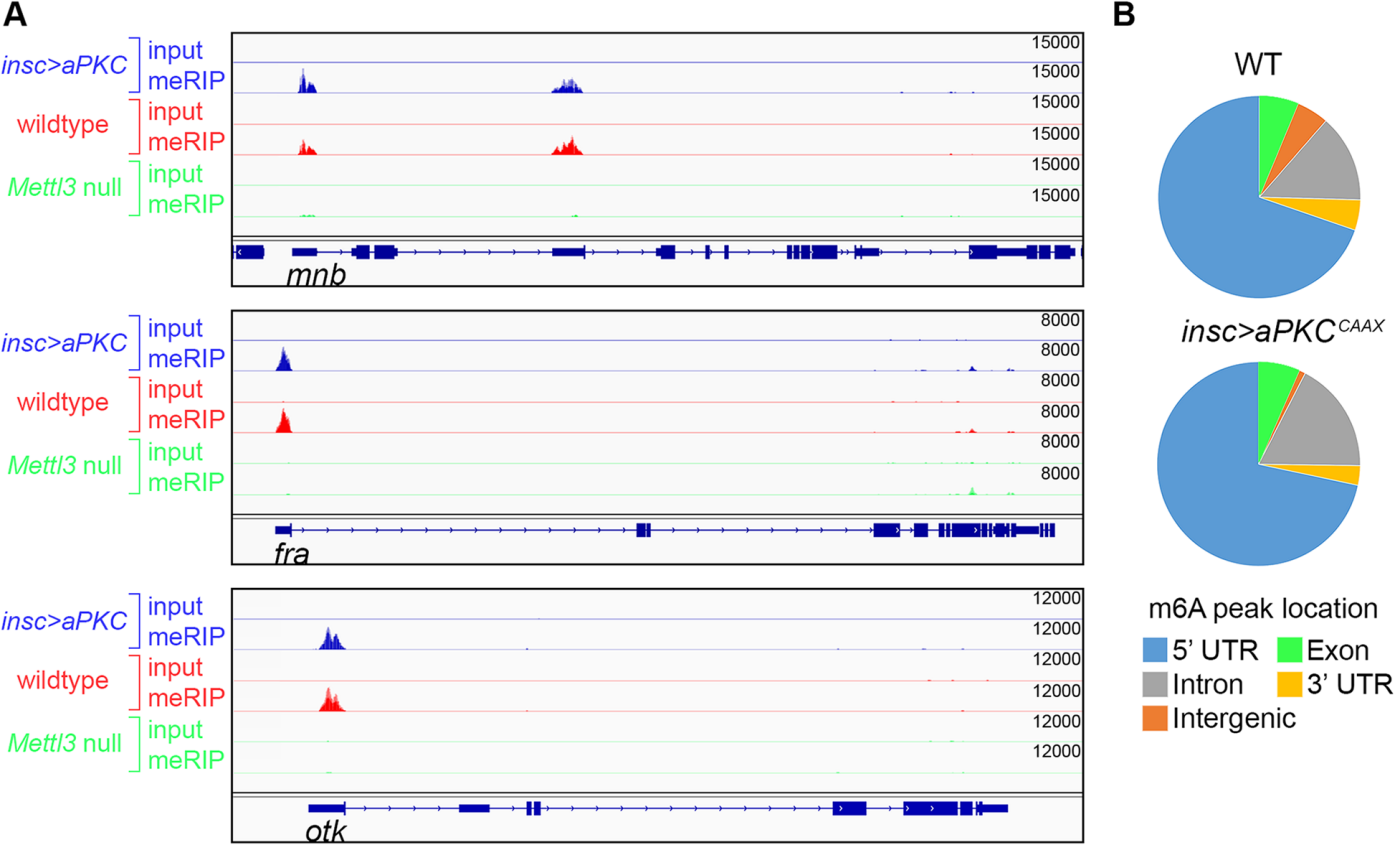
m6A peaks map to 5’ UTRs in neuroblast-biased and neuron-biased brains. **A** IGV plots of representative meRIP-seq data. Note that 5’UTR peaks are missing or significantly reduced in Mettl3 null brains while other peaks, for example in the downstream exons of fra, are independent of Mettl3. Such Mettl3-independent peaks were excluded from target identification and m6A position mapping. **B** Fraction of m6A peaks within different gene regions according to neuroblast-biased and neuron-biased meRIP

Subsequent meRIP-seq analysis of neuroblast-biased, neuron-biased and m6A-null RNA samples identified 867 m6A targets in the larval brain (Fig. 2A and Supplemental Table 1). 634 of these targets (73%) were also identified in adult *Drosophila* heads by Kan et al., revealing a high degree of m6A conservation across life cycle stages. As previously described, the m6A-null meRIP-seq data were useful for identifying “background” signal. This allowed high-confidence target identification and more accurate mapping of m6A peaks along a transcript: only peaks that were significantly enriched compared to m6A-null meRIP were included. Using this approach, we found that the vast majority of m6A peaks in the neuroblast-biased and neuron-biased transcriptomes map to the 5’ UTR (Fig. 2B). We used sequences from the combined neuron-biased and neuroblast-biased datasets to search for motifs associated with m6A and found significant enrichment of an AAACV motif. This motif contains the invariant AAAC core identified in other *Drosophila* m6A mapping studies [7, 9].

To gain insight into the potential roles of m6A in larval brain development, we used gene ontology analysis to identify functional categories overrepresented among m6A targets. This revealed significant enrichment of transcripts encoding regulators of essential neurodevelopmental processes, such as “synapse organization”, “dendrite development”, “neuroblast proliferation” and “neuron fate specification” in addition to processes known to be broadly important for development, such as “cell death”, “cytoskeleton organization”, and “Wnt signaling pathway” (Table 1). As expected, the combined profiling of neuroblast-biased and neuron-biased brains allowed identification of a large number of m6A targets (233 genes) that were not identified by previous m6A mapping in adult heads [9]. This novel set of m6A targets.

**Table 1:** Top 20 GO categories enriched among m6A targets, ranked by *p*-value. Count is the number of m6A targets in that category. Enrichment is the observed frequency of targets in that category (count / 867 total m6A targets) divided by the expected frequency (all genes in that category / total *Drosophila* genes)

GO Term	Definition	*P* value	Count	Enrichment
GO:0022008	neurogenesis	2.0 × 10^–112^	233	5.5
GO:0007154	cell communication	1.2 × 10^–92^	291	3.6
GO:0048812	neuron projection morphogenesis	1.0 × 10^–60^	125	6.1
GO:0006355	regulation of transcription	1.2 × 10^–46^	171	3.5
GO:0007611	learning or memory	3.1 × 10^–16^	41	5.5
GO:0,050,808	synapse organization	4.1 × 10^–15^	54	4.0
GO:0016358	dendrite development	4.9 × 10^–14^	41	4.9
GO:0008219	cell death	6.0 × 10^–13^	59	3.3
GO:0007010	cytoskeleton organization	6.5 × 10^–13^	79	2.7
GO:0042063	gliogenesis	9.8 × 10^–13^	28	6.7
GO:0008039	synaptic target recognition	1.4 × 10^–11^	20	9.3
GO:0008356	asymmetric cell division	2.7 × 10^–11^	28	6.0
GO:0007405	neuroblast proliferation	8.0 × 10^–11^	26	6.2
GO:0016055	Wnt signaling pathway	1.6 × 10^–10^	30	5.2
GO:0050795	regulation of behavior	1.5 × 10^–09^	26	5.5
GO:0007268	chemical synaptic transmission	1.1 × 10^–08^	45	3.2
GO:0030509	BMP signaling pathway	1.3 × 10^–07^	18	6.9
GO:0048665	neuron fate specification	2.6 × 10^–07^	13	10.1
GO:0000165	MAPK cascade	3.6 × 10^–06^	26	4.0
GO:0055057	neuroblast division	6.4 × 10^–05^	13	6.9

includes many genes known to regulate neuroblast proliferation, asymmetric cell division, neuron fate specification and axon pathfinding (Fig. 3A).

**Fig. 3:**
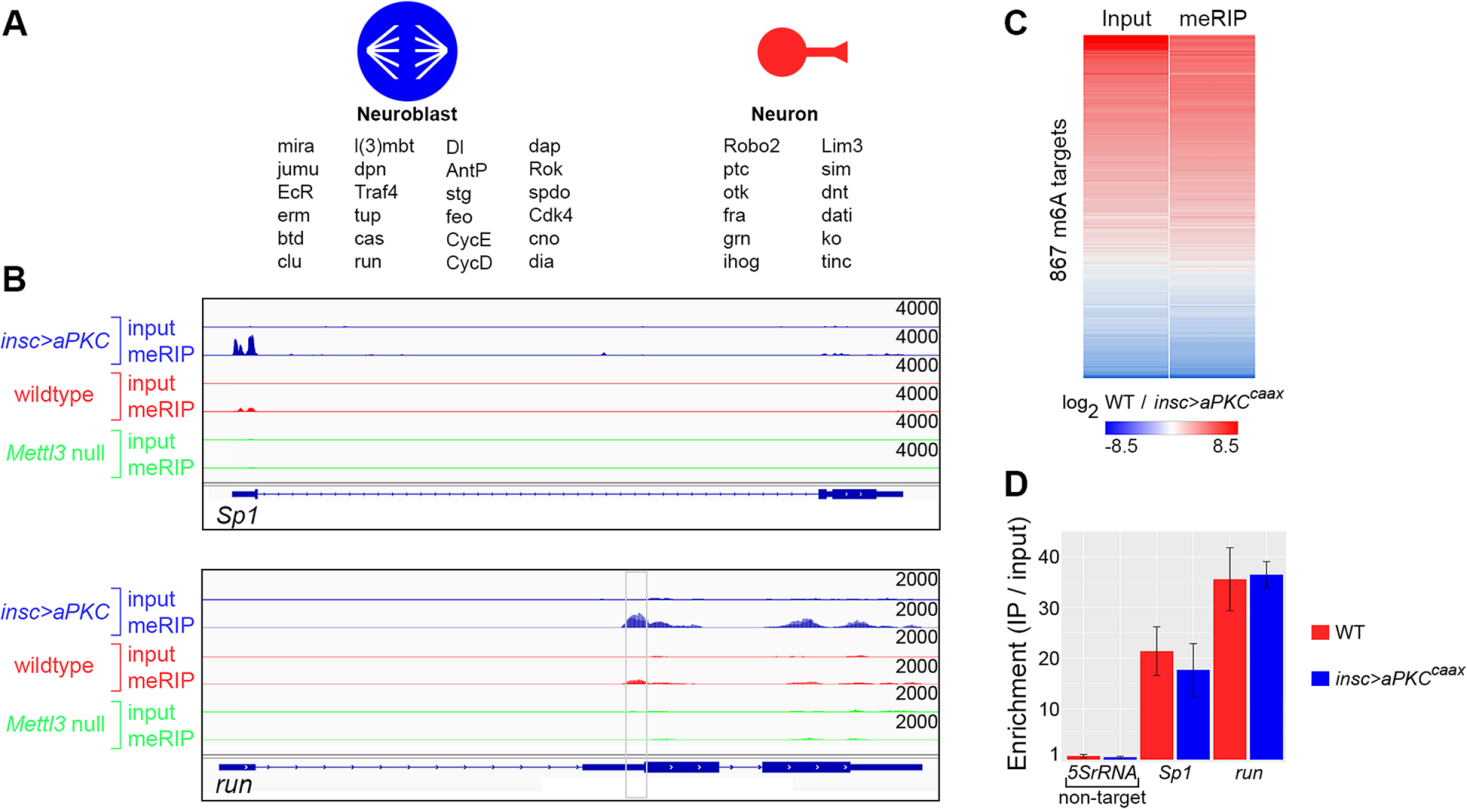
Novel m6A targets and evidence of uniform m6A stoichiometry between neuroblast-biased and neuron-biased brains. **A** Partial list of novel m6A targets identified in this study. Genes are listed below the cell type they are most associated with (cell cycle and fate determination genes are associated with neuroblasts, neuron identity and axon pathfinding genes are associated with neurons). **B** IGV plots of two genes with apparent increased m6A frequency in neuroblast-biased brains. A single Mettl3-dependent peak in the 5’UTR of run is outlined in gray. **C** Heat map comparing neuron-biased / neuroblast-biased (WT / insc > aPKCCAAX) ratios for all m6A targets based on input RNA-seq and meRIP-seq. **D** RT-qPCR of target transcripts in meRIP RNA versus input RNA. 5S rRNA is a negative control (not a m6A target). Data are average ± SEM for three independent input and meRIP samples

Comparing neuron-biased and neuroblast-biased meRIP-seq data revealed several genes with higher m6A peaks in one genotype or the other, potentially indicating cell type-specific differences in m6A stoichiometry (Fig. 3B). To test this possibility, we normalized meRIP-seq ratios (neuron-biased / neuroblast-biased) to input ratios (neuron-biased / neuroblast-biased). This identified cases where differential m6A peaks could be explained by differences in total transcript abundance. Following normalization for input reads and filtering for genes with statistically significant differences, we did not identify any evidence of differential m6A stoichiometry (Fig. 3C)*.* 135 genes had approximately equal input expression levels (fold-change ≤ 1.5 and no statistically significant difference between neuroblast-biased and neuron-biased input mRNA abundance), but none of these “uniformly” expressed transcripts showed evidence of elevated m6A frequency in neuroblast-biased or neuron-biased brains. This suggests that elevated m6A peaks in neuroblast-biased brains, as shown for *Sp1* and *run* in Fig. 3B, are due to elevated expression of the corresponding transcripts in neuroblasts. The converse is true for elevated m6A counts in neuron-biased brains. We further tested this conclusion using m6A immunoprecipitation and RT-qPCR of *Sp1* and *run* (Fig. 3D). 5S rRNA served as a negative control in these experiments as it was not identified as a m6A target in our experiments and is known to lack methyladenosine in metazoans [23]. meRIP and RT-qPCR confirmed *Sp1* and *run* as m6A targets and ruled out differential m6A between neuroblast-biased and neuron-biased brains. Overall, our m6A mapping indicates that m6A is selectively targeted to neurodevelopmental genes in neuroblasts and neurons and that for transcripts present in both cell types, the degree of m6A modification is largely constant.

### m6A correlates with low translation efficiency and low mRNA stability

**Fig. 4:**
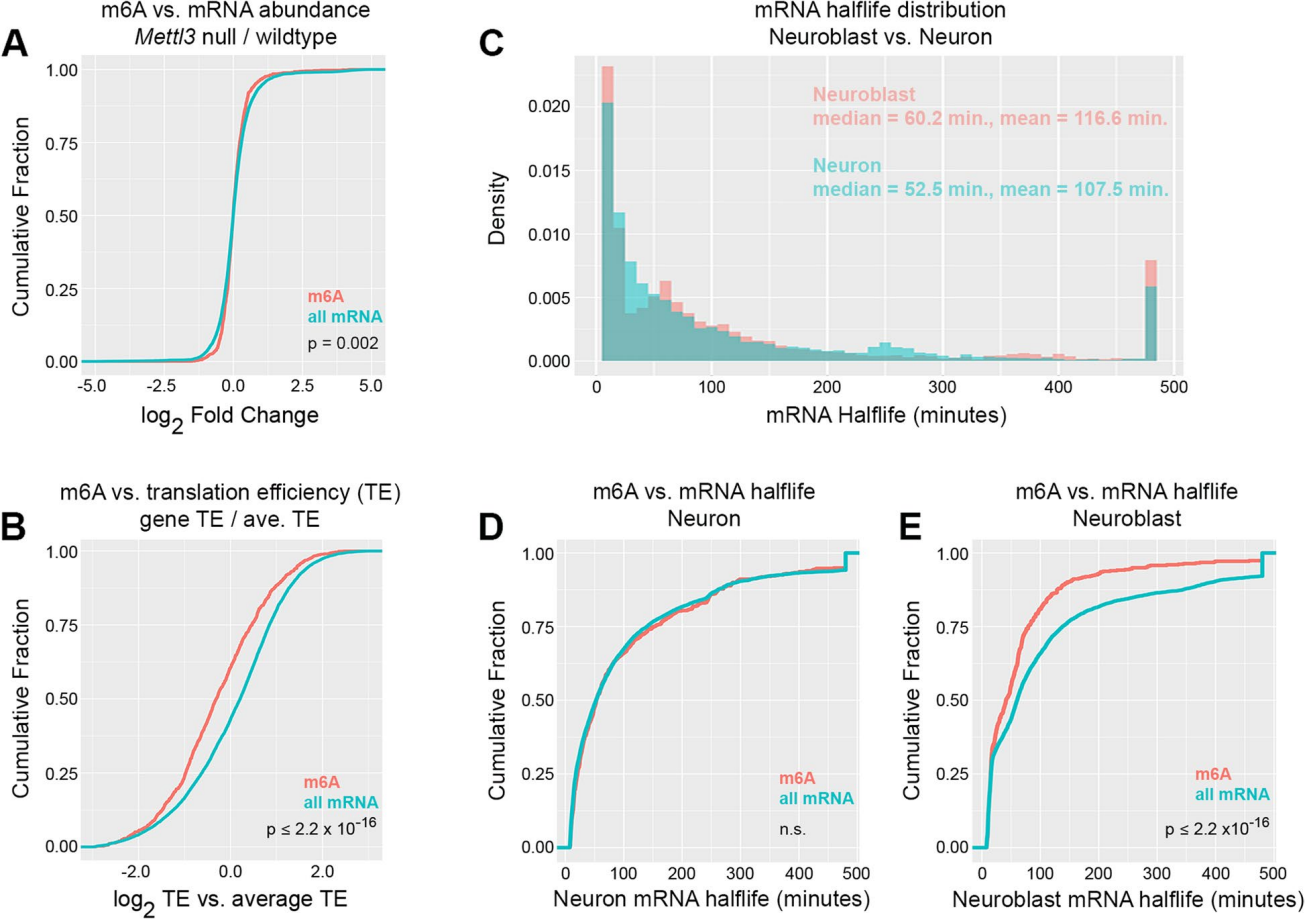
m6A correlates with low translation efficiency and low mRNA stability in neuroblasts. **A** Loss of m6A does not significantly affect target mRNA abundance. Log2 fold-change in transcript abundance in Mettl3 null brains versus wildtype brains, plotted as the cumulative distribution of m6A targets compared to all larval brain transcripts. **B** m6A correlates with low translation efficiency (TE). Log2 relative TE (transcript-specific TE / average TE), plotted as the cumulative distribution of m6A targets compared to all larval brain transcripts with matching adult head TE data. **C** Distribution of mRNA half-lives in neuroblasts and neurons, as determined by EC-tagging pulse-chase. Half-life values greater than 480 min were rounded down to 480 min. **D** and **E** m6A correlates with low mRNA half-life in neuroblasts but not neurons. mRNA half-life plotted as the cumulative distribution of m6A targets compared to all mRNAs as measured in neurons (**D**) or neuroblasts (**E**). P-values were determined by two sided Kolmogorov–Smirnov tests

Given that m6A has been implicated in a range of mRNA metabolic processes, we next sought clues to the molecular function of m6A during larval brain development. Akhtar et al. identified a role for m6A and the nuclear m6A reader in enhancing transcription by relieving RNAP II pausing at target genes. This was demonstrated in *Drosophila* S2 cells and the phenomena has not been described in vivo or in a developmental context. To test this possible function, we used RNA-seq measurements of total mRNA abundance from wildtype brains and *Mettl3* null brains. We reasoned that if m6A significantly enhances transcription in larval brains, the absence of m6A would result in decreased target abundance due to increased RNAP II pausing. As previously shown for adult *Drosophila* heads [9], this analysis failed to identify a strong directional relationship between m6A and transcript abundance (Fig. 4A). We also tested for a relationship between m6A and translation efficiency (TE). Using the adult head ribosome profiling data analyzed by Kan et al. [9], we found a similar significant enrichment of m6A in mRNAs with low translation efficiency (Fig. 4B).

Next, we tested for any relationship between m6A and mRNA stability. We obtained mRNA half-life measurements for neural progenitors and neurons using EC-tagging pulse-chase [14]. Briefly, this approach uses targeted expression of a cytosine deaminase-uracil.

phosphoribosyltransferase (CD:UPRT) fusion enzyme to convert 5-ethynylcytosine (EC) into 5-ethynyluridine (EU)-monophosphate in specific cell types. EU is incorporated into nascent mRNAs of target cells and the tagged RNAs can be purified after “pulse” feeding 5EC and at subsequent “chase” timepoints in which excess uridine is provided to ensure no new tagged transcripts are made. We used *insc-Gal4* to express *UAS-CD:UPRT* in neural progenitors and *nSyb-Gal4* to express *UAS-CD:UPRT* in neurons. Globally, neural progenitor and neuron transcriptomes had similar half-life distributions (Fig. 4C and Supplemental Table 2), indicating that transcriptome-wide mRNA decay kinetics do not significantly differ between neural progenitors and neurons. However, differences were revealed when we analyzed the half-lives of m6A targets: there was no relationship between m6A and mRNA stability in neurons (Fig. 4D), while m6A targets were significantly less stable in neuroblasts (Fig. 4E).

**Fig. 5:**
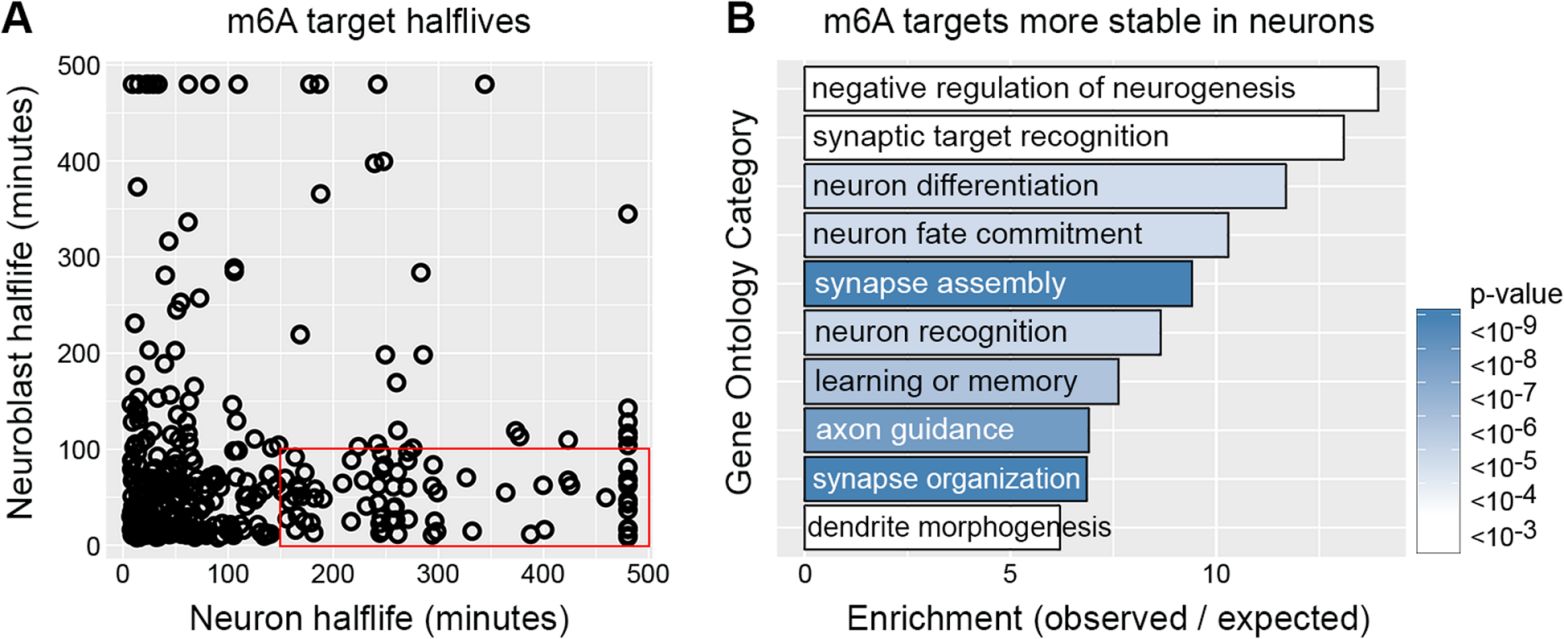
m6A targets encoding regulators of neuron-biased functions are stabilized in neurons. **A** m6A target half-life in neuroblasts compared to neurons. An example of neuron-stabilized transcripts (half-life ≤ 100 min in neuroblasts and ≥ 150 min in neurons) are outlined by a red box. **B** Gene ontology categories significantly enriched among m6A targets that are ≥ 1.5-fold more stable in neurons

To further investigate the different relationships between m6A and stability in neuroblasts and neurons, we directly compared the half-lives of m6A targets in each cell type and found that 185 m6A targets are at least 1.5-fold more stable in neurons (Fig. 5A). If one assumes m6A directly affects mRNA stability, this differential decay is surprising given that our data suggest m6A is constant between neuroblasts and neurons. Differential stability could be caused by varied Ythdf expression, however; prior transcriptome profiling of purified neuroblasts and neurons found that Ythdf mRNA is present at equally high levels in progenitors and neurons [24]. Alternatively, these data agree with a model in which the difference between neuroblasts and neurons is due to m6A-independent stabilization of target mRNAs in neurons. GO analysis of the neuron-stabilized m6A targets revealed enrichment of transcripts involved in neuron-specific functions such as “synapse assembly”, “dendrite development” and “axon guidance” (Fig. 5B), supporting the model that these transcripts are likely selectively stabilized to support the needs of mature neurons. We conclude that neuron-specific stabilization of m6A targets explains the lack of correlation between m6A and half-life in neurons.

### m6A and Ythdf enhance target protein expression in larval brains

The analyses described above reveal correlations between m6A, mRNA translation and mRNA decay, but these findings do not reveal underlying mechanisms or causal relationships. With respect to translation, two mechanisms have been described in *Drosophila*: translation inhibition that requires Fmr1 [7] and Ythdf-dependent translation enhancement [9]. Comparing our m6A targets with previously identified m6A-dependent Fmr1 targets in the larval central nervous system revealed an overlap of only 5.8% (50 genes). Since the majority of our targets are not predicted to be regulated by Fmr1, we conclude that the translation enhancing effect may be more relevant. With respect to mRNA stability, 3’ UTR m6A in mammalian transcripts induces decay via DF proteins recruiting the CCR4-NOT deadenylase complex [5] but a decay pathway triggered by 5’UTR m6A has not been described in any species. Instead, we predict that the relationship between m6A and mRNA is indicative of a compensatory mechanism, similar to that described for translation efficiency. In this case, we predict that 5’UTR m6A enhances translation of low stability transcripts whose decay is regulated by m6A-independent mechanisms.

According to the translation enhancement model, *Mettl3* deletion should decrease target protein production and *Ythdf* overexpression should increase target protein production. To test this model in the developing larval brain, we performed quantitative immunofluorescent imaging of proteins encoded by m6A targets in wildtype brains, *Mettl3* null brains and *Ythdf* overexpressing brains (overexpressing *Ythdf* in neural progenitors using *insc-Gal4* > *UAS-Ythdf*). We measured immunofluorescent signal for two m6A targets, the transcription factor Runt (Run) and the cell cycle regulator Cyclin D (CycD), in addition to one non-target, the transcription factor Asense (Ase). Translation efficiency data are not available for *run* and *ase*, likely because these genes are not expressed or are only expressed at low levels in adult brains, but the TE value for *CycD* in adult heads is 1.17 compared to an average value of 1.37 [25]. In contrast to the TE data, mRNA stability data are available for each of these genes. In neural progenitors *run* decays very rapidly (half-life of 5.1 min) and is more stable in neurons (half-life of 17.6 min). In larval brains, *CycD* and *ase* expression is primarily restricted to neural progenitors and we therefore only obtained progenitor-specific decay measurements for these transcripts: *CycD* has a half-life of 136.3 min and *ase* has a half-life of 16.1 min.

**Fig. 6:**
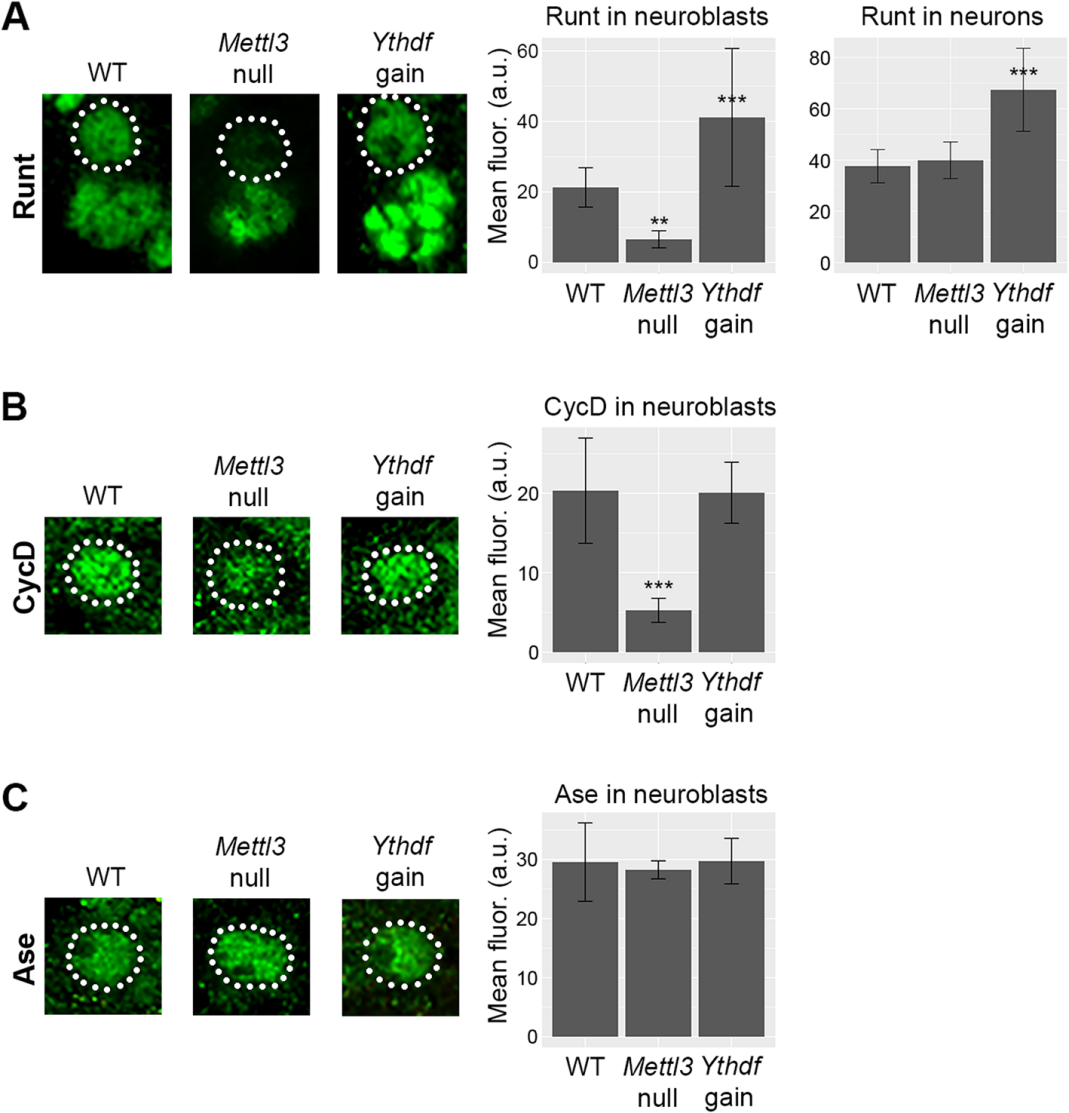
m6A and Ythdf increase target protein abundance in neuroblasts and neurons. **A** Representative images of Runt in neuroblasts (outlined by white dotted line) and neurons (cells clustered below neuroblast) in wildtype, Mettl3 null and Ythdf overexpressing brains. The fluorescent signal intensity (mean and standard deviation) for Runt in each genotype and cell type is shown at right. **B** Representative images of CycD in neuroblasts (outlined by white dotted line) in wildtype, Mettl3 null and Ythdf overexpressing brains. CycD was not detected in neurons. The fluorescent signal intensity (mean and standard deviation) for CycD in each genotype is shown at right. **C** Representative images of Ase in neuroblasts (outlined by white dotted line) in wildtype, Mettl3 null and Ythdf overexpressing brains. Ase was not detected in neurons. The fluorescent signal intensity (mean and standard deviation) for Ase in each genotype is shown at right. All fluorescent intensity measurements are derived from analysis of ≥ 20 cells from ≥ 6 different brain lobes. Statistical significance was determined by one way ANOVA followed by Tukey post-test. *P*-values: ** = 1 × 10–4, *** ≤ 1 × 10–7

Runt expression in neuroblasts changed in a manner corresponding to the translation enhancement model: Runt signal decreased in *Mettl3* null neuroblasts and increased in *Ythdf* overexpressing neuroblasts (Fig. 6A). In neurons, Runt signal was unaffected by loss of *Mettl3* but increased in *Ythdf* overexpressing brains. Similar to Runt, CycD protein levels decreased in *Mettl3* null neuroblasts, but *Ythdf* overexpression did not alter CycD abundance (Fig. 6B). Finally, as expected, neither *Mettl3* loss-of-function or *Ythdf* overexpression altered.

the abundance of the non-target Asense (Fig. 6C). The *run* and *CycD* data support our prediction that m6A does not induce mRNA decay; if this were the case, *Mettl3* deletion would.

most likely increase protein levels (we observe the opposite effect) and *Ythdf* overexpression would decrease protein levels (again, we see the opposite). Instead, these results support the model that 5’UTR m6A enhances translation of target mRNAs in the developing nervous system.

## Discussion

Precise deployment of genetic information during neurogenesis requires multiple layers of post-transcriptional control. m6A provides one such layer, but the full diversity of cell types and pathways affected by m6A, and the degree to which m6A modification and target metabolism are dynamically regulated, is not fully understood. We investigated these questions of m6A dynamics in the context of *Drosophila* larval brain development. The m6A profiles we obtained from neuroblast-biased and neuron-biased brains expand the list of known m6A targets in the *Drosophila* nervous system, contributing to a deeper understanding of m6A targeting during neurodevelopment. Importantly, our results lend support to the model that m6A stoichiometry of individual transcripts is largely uniform and does not vary according to cell type. In spite of this uniformity, we show that m6A targets may be metabolized in a cell type-specific manner, particularly if target mRNA processing pathways vary by cell type. Finally, we provide neural-specific in vivo evidence to support the translation enhancement model proposed by Kan et al. [9]. Altogether our results point to m6A as an important modifier of protein output from key neurodevelopmental transcripts.

While *insc* > *aPKC*^*CAAX*^ brains are not exclusively composed of neuroblasts and wildtype brains are not exclusively composed of neurons, the transcriptomes of each are heavily biased toward one cell type or the other and have a high likelihood of revealing differential m6A stoichiometry. However, no significant differential m6A targeting was indicated by our analyses. This outcome agrees with the theory that differential m6A stoichiometry is rare [4]. Part of this theory is based on the mechanics of m6A deposition and removal; the enzymes that write and erase m6A appear to be ubiquitous and it is unclear how their activity might be conditionally modified to alter only a subset of targets. In the context of *Drosophila* neural differentiation, dynamic m6A targeting would require selective alteration of methyltransferase activity between neuroblasts and neurons in a way that targeted specific genes, or transcript-specific demethylase activity in one cell type versus the other. While such mechanisms may exist and could involve differences in RNAP II pausing at target genes, we interpret our results as supporting the “non-dynamic m6A” model, at least along the neural differentiation axis in *Drosophila*.

In addition to identifying novel m6A targets, we also obtained transcriptome-wide mRNA decay measurements in neural progenitors and neurons. A link between m6A and mRNA decay in *Drosophila* was previously ruled out by comparing adult head m6A targets and embryonic central nervous system mRNA half-lives. A limitation of this prior analysis is that the embryo mRNA decay data were mainly derived from neurons; neural progenitor-specific measurements were missing. Our cell type-specific mRNA half-life data revealed a correlation between m6A and short half-life in neuroblasts but no correlation between m6A and half-life in neurons. It is important to recognize that our m6A – mRNA decay results demonstrate a correlation (or lack thereof) and not causation. Given that m6A stoichiometry is constant between neuroblasts and neurons, that the Ythdf reader is expressed at equal levels in both cell types [24], and that a molecular pathway linking 5’ UTR m6A to mRNA decay is not known, we interpret these results as evidence of m6A-independent stabilization of target transcripts in neurons. Neuron-specific stabilization of m6A targets may occur via canonical mRNA decay pathways. For example, loss of a destabilizing RNA-binding protein during differentiation could increase target mRNA stability in neurons relative to neuroblasts. In this scenario, increased stability would synergize with the translation enhancing effect of m6A to optimize expression of proteins necessary for post-mitotic functions like synapse assembly. Such a mechanism aligns with the concept that m6A is a modifier of protein output from target transcripts but not the main driver of target mRNA metabolism.

A major question in developmental biology is how varying rates of transcription, decay and translation combine to determine gene expression dynamics. Short mRNA half-life and inefficient translation favor low protein output, but the m6A pathway may have evolved to fine-tune protein levels of targets with these properties. For example, rapid decay of *run* in neuroblasts is expected to result in very low protein levels. m6A-dependent enhancement of *run* translation could increase the output of each transcript prior to degradation and may help achieve expression levels appropriate for Runt activity in neuroblasts. Our quantitative imaging of Runt in neuroblasts supports this model: Runt levels decrease in *Mettl3* null brains and increase in *Ythdf* overexpressing brains. *Runt* mRNA half-life increases threefold in neurons and there is a corresponding increase in Runt signal in neurons compared to neuroblasts. Loss of *Mettl3* in neurons does not result in a quantifiable decrease in Runt levels, perhaps because neuron-specific stabilization of *run* mRNA compensates for the loss of m6A. Surprisingly, *Ythdf* overexpression in neural progenitors significantly increased Runt signal in neurons. This may be due to elevated Runt production in progenitors and excess Runt being actively or passively inherited by neurons. Alternatively, Ythdf itself may be inherited by neurons where it is sufficient to increase Runt production. While *Mettl3* loss-of-function decreased CycD signal in neuroblasts, *Ythdf* overexpression had no effect. This may indicate a role for m6A position in affecting translation: the largest *Mettl3*-dependent peak in *run* is concentrated near the start codon, while *CycD* has two Mettl3-dependent peaks distributed more broadly over the 5’ UTR (data not shown). Whether m6A position along a transcript determines the degree to which Ythdf enhances translation remains to be determined.

Our finding that m6A is targeted to neurodevelopmental regulatory genes in neuroblasts and neurons raises the question of how target specificity is achieved. A recently described targeting mechanism in *Drosophila* provides an intriguing answer that could also explain the relationships between m6A, mRNA half-life and translation efficiency. In *Drosophila*, the m6A methyltransferase complex is selectively recruited to promoters where RNA polymerase II is bound in a paused, non-elongating state [12]. It is well established that genes involved in developmental transitions and dynamic cellular processes have high levels of paused RNAP II in *Drosophila* [12, 26, 27]. Additionally, we and others have shown that transcripts involved in developmental transitions and dynamic cellular processes tend to have short half-lives [28, 29], and in many instances those transcripts become more stable in neurons [30]. Finally, transcripts encoding developmental regulators are also known to contain sequence features like uORFs [25] or secondary structures [31] that influence translation efficiency. The fact that genes encoding developmental regulators are transcriptionally-regulated by paused RNAP II (the signal for m6A methylation) and are post-transcriptionally regulated via dynamic mRNA decay and translation provides a parsimonious explanation for the m6A – mRNA decay – TE relationships we identified.

## Conclusions

This work expands our understanding of the role of m6A in neural development by providing a detailed view of m6A targeting and target metabolism in neural progenitors and neurons. The use of neuroblast-biased brains allowed identification of m6A targets missed by prior profiling efforts and allowed comparison of m6A stoichiometry between neuroblast-biased and neuron-biased transcriptomes. We found that there is little variation in m6A stoichiometry between these transcriptomes. Our neuroblast and neuron mRNA half-life data revealed a strong correlation between m6A and low mRNA stability in neuroblasts but not neurons. We conclude that the lack of correlation in neurons is due to m6A-independent stabilization of those targets, in accordance with evidence that 5’UTR m6A in *Drosophila* affects translation and not stability. Finally, we provide neural-specific in vivo evidence to support the translation enhancement model. Overall, our findings contribute to the view that m6A is important for fine-tuning gene expression during neural development and that dynamic changes in m6A stoichiometry are rare.

## Supplementary Information

**Additional file 1: Supplemental Table 1.** Neuroblast and neuron m6A targets. 

**Additional file 2: Supplemental Table 2.** Neuroblast and neuron mRNA half-lives.

## Data Availability

RNA-seq data underlying the results presented in this study are available from the NCBI Gene Expression Omnibus (series records GSE214058 and GSE214062).

## Abbreviations

m6A: N6-methyladenosine
3’UTR: 3-Prime untranslated region
5’UTR: 5-Prime untranslated region
ALH: After larval hatching
meRIP: Anti-m6A RNA immunoprecipitation
RNA-seq: RNA sequencing
meRIP-seq: RNA sequencing of anti-m6A immunoprecipitated RNA
RT-qPCR: Reverse transcription of RNA followed by quantitative polymerase chain reaction
rRNA: Ribosomal RNA
mRNA: Messenger RNA
WT: Wildtype
RNAPII: RNA polymerase II
TE: Translation efficiency
EC: 5-Ethynylcytosine
GO: Gene ontology
uORF: Upstream Open Reading Frame
